# Comment on: “The role of SIGLEC9 in immunosuppression and prognosis in cervical cancer”

**DOI:** 10.1016/j.clinsp.2026.100953

**Published:** 2026-05-11

**Authors:** Herik Fernande de Oliveira, Francisco Dantas de Souza

**Affiliations:** Universidade de São Paulo (USP), São Paulo, SP, Brazil

Dear Editor,

We carefully reviewed the recent article by Wang et al., which investigates the role of SIGLEC9 in shaping the tumor immune microenvironment and its prognostic relevance in cervical cancer. By integrating bioinformatic analyses with tissue-based validation, the authors contribute meaningful data to the expanding literature on the sialic acid–SIGLEC axis as an emerging immune checkpoint pathway in oncology.^1^

In the context of these findings, a few methodological aspects merit further consideration to help contextualize the interpretation of the results. First, the dichotomous classification of tumor-associated macrophages into M1 and M2 subsets, particularly when inferred from bulk transcriptomic deconvolution algorithms, may oversimplify the well-established plasticity of TAMs. Current evidence supports the concept that macrophage activation states exist along a functional continuum rather than discrete phenotypes, which may limit the biological resolution of M1/M2-based interpretations.^2^^,^^3^

Second, although statistically significant associations between SIGLEC9 expression and overall survival were observed, the relatively limited sample size and number of outcome events may affect the robustness of prognostic inferences. Validation in larger, independent cohorts would therefore be important to confirm the reproducibility of SIGLEC9 as a prognostic biomarker, as has been demonstrated in other tumor types where SIGLEC9⁺ tumor-associated macrophages correlate with adverse clinical outcomes.^4^

Third, stratification based on median SIGLEC9 expression, while methodologically convenient, may not reflect biologically or clinically optimal thresholds. Alternative strategies, such as outcome-driven cutoffs or continuous-variable modeling, could offer a more nuanced assessment of risk across expression levels.

Finally, while the association between SIGLEC9 expression and an immunosuppressive tumor microenvironment is compelling, the absence of functional experiments limits the ability to distinguish whether SIGLEC9 acts as an active driver of immune dysfunction or represents a surrogate marker of an already established suppressive milieu. This distinction is particularly relevant given preclinical evidence showing that disruption of the sialic acid-SIGLEC9 axis can restore immune effector function and enhance responses to immunotherapy.^5^^,^^6^

Taken together, these considerations suggest that the translational implications of SIGLEC9 in cervical cancer should be regarded as hypothesis-generating rather than practice-changing. Nonetheless, the work by Wang et al. provides a strong rationale for future functional and prospective studies exploring whether therapeutic targeting of SIGLEC9 could synergize with established PD-1/PD-L1 blockade, particularly in patients with immunologically “cold” or checkpoint-resistant tumors.

## Data availability

The datasets generated and/or analyzed during the current study are available from the corresponding author upon reasonable request.

## Conflicts of interest

The authors declare no conflicts of interest.
